# Decision-Making by Handball Referees: Design of an *ad hoc* Observation Instrument and Polar Coordinate Analysis

**DOI:** 10.3389/fpsyg.2017.01842

**Published:** 2017-10-20

**Authors:** Juan P. Morillo, Rafael E. Reigal, Antonio Hernández-Mendo, Alejandro Montaña, Verónica Morales-Sánchez

**Affiliations:** Departamento de Psicología Social, Trabajo Social, Antropología Social y Estudios de Asia Oriental, Facultad de Psicología, Universidad de Málaga, Málaga, Spain

**Keywords:** refereeing, handball, polar coordinates, decision-making, systematic observation

## Abstract

Referees are essential for sports such as handball. However, there are few tools available to analyze the activity of handball referees. The aim of this study was to design an instrument for observing the behavior of referees in handball competitions and to analyze the resulting data by polar coordinate analysis. The instrument contained 6 criteria and 18 categories and can be used to monitor and describe the actions of handball referees according to their role/position on the playing court. For the data quality control analysis, we calculated Pearson's (0.99), Spearman's (0.99), and Tau Kendall's (1.00) correlation coefficients and Cohen's kappa (entre 0.72 y 0.75) and Phi (entre 0.83 y 0.87) coefficients. In the generalizability analysis, the absolute and relative generalizability coefficients were 0.99 in both cases. Polar coordinate analysis of referee decisions showed that correct calls were more common for central court and 7-meter throw calls. Likewise, calls were more likely to be incorrect (in terms of both errors of omission and commission) when taken from the goal-line position.

## Introduction

Referees have a key role in elite sports competitions (Cruz, 1997; Dohmen and Sauermann, 2015). Officiating a match between two teams is a difficult task that is further complicated by the need to take decisions on a range of events that occur within a short space of time (Plessner, 2005; Mascarenhas and Smith, 2011). The decisions taken by referees can influence the unfolding of events during a match and even decide the outcome (Philippe et al., 2009). It is therefore important to analyze and improve referee performance in these contexts.

Good referees share certain qualities. They must be very knowledgeable about the rules of the game, have a good level of physical fitness, position themselves correctly on the court or pitch, have good visual and auditory acuity, and be highly motivated and capable of taking on-the-spot decisions and controlling their emotions (Weinberg and Richardson, 1990; Mascarenhas et al., 2005; Simmons, 2011). These qualities, can, however, be modified by various factors that can affect decision-making processes (Weston et al., 2012), such as previous experiences with teams and/or players and even player reputation and gender.

Refereeing in team handball is a complex task, as handball is a fast, physical game involving continuous contact and offensive and defensive actions (Souchon et al., 2009). To meet the demands of officiating a match and withstand the pressure generated by players, crowds, and critical moments, referees need to be sufficiently prepared, both psychologically and technically (Gimeno et al., 1998; Debanne, 2014). Insufficient preparation can lead to attention and concentration difficulties, doubts regarding decisions, increased anxiety levels, and a greater risk of making mistakes (Estrada and Pérez, 2008; Debanne, 2014).

Decision-making by handball players has received considerable attention in recent years, and numerous tools have been created to analyze what occurs in game situations (Luckwü and Guzmán, 2011; Martín et al., 2013; Loffing et al., 2015; Weigel et al., 2015; Helm et al., 2016). Tools have also emerged to analyze the activity of coaches, who have an important influence on match tactics and outcomes (Debanne and Fontayne, 2009; Debanne, 2014). There is, however, a need for reliable, accurate tools for analyzing the performance of handball referees, as very few have been developed (Souchon et al., 2009).

Research in this area has sought to identify different elements that can help to interpret decision-making in sport (Araujo et al., 2016). Systematic observation, for instance, offers a range of techniques for analyzing behavior in natural settings (González et al., 2013; Lapresa et al., 2013; Anguera and Hernández-Mendo, 2014; Sousa et al., 2014). Numerous studies have shown that observational methodology is an adequate methodology for analyzing behavior in sport (Anguera and Hernández-Mendo, 2013). It is (a) non-intrusive, (b) has a high level of ecological validity (i.e., it analyzes natural behaviors in natural settings), and (c) offers high analytical specificity through the construction of *ad hoc* observation instruments designed specifically for analyzing specific game situations in the environment in which they occur (Araujo, 2011, 2013; Pinder et al., 2011).

One technique that has shown great potential in this area in recent years is polar coordinate analysis (Sackett, 1980). It is among the most informative techniques (Araujo et al., 2016) and is particularly powerful when the concept of genuine retrospectivity is applied (Anguera, 1997). Recent years have seen a rapid uptake of polar coordinate analysis in the field of Sports Sciences, where it has been used to analyze a range of sports, including soccer, tennis, and handball (Castellano et al., 2007; Perea et al., 2012; Morillo and Hernández-Mendo, 2015; Morillo et al., 2015; Castañer et al., 2016; López et al., 2016; Santoyo et al., 2017; Tarragó et al., 2017).

To analyze decisions taken in sport, it is necessary to analyze the different actions that occur during a game (Pinder et al., 2011). Polar coordinate analysis is a suitable technique for identifying and helping to understand these actions. Prudente et al. (2017), for example, used this technique to show how playing time influenced tactical decisions made by handball players. Polar coordinate analysis has also been used in beach volleyball to identify erroneous behaviors in relation to passes and receptions (Morillo et al., 2015). Finally, the technique has been successfully applied to analyzing tactical decisions taken in track events.

Polar coordinate analysis is a powerful technique that reduces the volume of data to be processed without losing important information. It is used to identify significant relationships between a behavior of interest, known as the focal behavior, and other behaviors, known as conditional behaviors, and presents these in an easy-to-interpret vector format (Hernández-Mendo and Anguera, 1998; Anguera and Losada, 1999; Gorospe and Anguera, 2000). The technique involves using adjusted residuals derived from sequential analysis (z scores) to calculate Z_*sum*_ statistics (Z_*sum*_ = ∑z/√n) (Cochran, 1954). This computation is possible, as both the frequency of the focal behavior (n) and the Z scores for each of the lags considered are known. These Z scores are independent of each other, as they are computed using the binomial test, which compares observed probabilities (corresponding to textual units derived from observation of the teachers' discourse) with expected probabilities (chance occurrences). The relationship between the focal behavior and the conditional behaviors is estimated using the angle of the resulting vector, while the strength is estimated using the vector radius (Anguera et al., 1997; Castellano and Hernández-Mendo, 2003). A crucial component of polar coordinate analysis is that its powerful data reduction feature permits the consideration of both retrospective and prospective perspectives. In other words, it shows what happens before and after the behavior of interest.

Given the scarcity of tools available for analyzing the activity of handball referees, the main aim of this study was to design a tool that could be used to objectively analyze referee behavior and performance in competition situations. A second aim was to test the tool using data from three matches at the 2013 World Men's Handball Championship held in Spain.

## Materials and methods

As we used an observation instrument that combined field formats with category systems, the observational design was multidimensional (Morillo et al., 2015; Prudente et al., 2017). The specific design was follow-up/idiographic/multidimensional, which fits into quadrant I of the systematic observation designs described by Anguera et al. (2011).

### Participants

A group of six observers, all male referees who officiated regional handball matches in Andalusia, Spain, participated in the data quality control phase. They were aged between 22 and 26 years (mean = 23.50; *SD* = 1.26) and had between 5 and 8 years' refereeing experience. For this analysis, the observers studied the semi-final between Spain and Slovenia at the 2013 World Men's Handball Championship.

The polar coordinate analysis was performed using data coded by a single observer from three final-stage matches in the same championship: the semi-final between Spain and Slovenia, the semi-final between Denmark and Croatia, and the third place game between Slovenia and Croatia.

The ethical requirements of observational methodology were applied to the current study and performed in accordance with the ethical standards laid down in the Declaration of Helsinki.

### Instruments

The observation instrument used to analyze and code the referees' actions in the matches analyzed was designed within the framework of observational methodology (Anguera, 1990, 1991; Anguera and Hernández-Mendo, 2014). Given the scarcity of existing theoretical constructs and the multidimensional nature of handball, the coding system (observation instrument) was built using an empirical-inductive approach (Castellano et al., 2000; Morillo et al., 2015).

The instrument comprised a combination of a field format system for each criterion (Anguera, 1979; Anguera and Hernández-Mendo, 2013) and a system of exhaustive, mutually exclusive categories. The final instrument contained 6 criteria and 18 categories (Table 1).

**Table 1:** Observation instrument: Criteria and corresponding categories and codes.

**Criterion**	**Category**
Position of referee	POS_CEN: center of court POS_FON: goal line
Responsibility: Whether or not the call that was made or should have been made was the responsibility of the referee being observed	PER_SI: Yes, the call was his responsibility PER_SI: No, the call was not his responsibility
Whistle: Whether or not the whistle was blown	PIT_SI: Yes, the whistle was blown PIT_NO: No, the whistle was not blown
Decision (responsibility)	MDO_ACI: The call was correct and was the responsibility of the referee being observed MDO_ERO: Error of omission; the call was the responsibility of the referee being observed but was not made MDO_ERC: Error of commission; the call was not the responsibility of the referee being observed but was made
Type of infraction	TIP_TECGF: Technical, free throw TIP_TEC7: Technical, 7-meter throw foul TIP_DIS: Warning, progressive punishment, direct dismissal, disqualification or disqualification with written report TIP_T-D: Technical foul-punishment; free throw or 7-meter throw fouls that include a personal punishment TIP_T-T: Technical-tactical; loss of possession without punishment TIP_TTD: Technical-tactical with punishment; loss of possession with punishment
Decision (action)	MDA_ACI: Correct call MDA_ERR: Incorrect call

For the polar coordinate analysis, we chose three focal behaviors that would permit analysis of individual interventions by referees, as this is a key aspect of refereeing. The categories chosen were related to decision (responsibility). Although the other categories are also important, we chose the three categories that could provide the most useful information for the aim of the study. These were:

MDO_ACI: Correct call by referee responsible for making the call,
MDO_ERO: Failure to make a call by referee responsible for the call,
MDO_ERC: Call made by referee not responsible for making it.

The data were coded and analyzed using HOISAN (Hernández-Mendo et al., 2012, 2014), a software program that performs polar coordinate analysis and presents the output in the form of vector maps. The generalizability analysis was performed in SAGT (Hernández-Mendo et al., 2016).

### Procedure

A generalizability analysis was used to test the validity and accuracy of the *ad hoc* observation instrument (Blanco-Villaseñor et al., 2000, 2014; Castellano et al., 2000). Generalizability coefficients provide an estimate of how the observed mean compares with the mean of all possible observations (Blanco-Villaseñor et al., 2000, 2014). Inter-observer agreement was assessed to estimate reliability (Anguera, 1990; Morillo and Hernández-Mendo, 2015).

For the data quality control analysis, three different moments of the semi-final between Spain and Slovenia were analyzed by previously trained observers. Two of the moments were observed by the same team of observers and the third one was observed by a second team. To maximize inter-observer agreement, the observers were trained (Morillo and Hernández-Mendo, 2015) and provided with a purpose-designed observation protocol. In addition, the data were coded using the consensus agreement method described by Anguera (1990). Cohen's kappa coefficients, generalizability analysis, and correlation coefficients were used to measure intra- and inter-observer agreement; the results in all cases were higher than 0.90. In the subsequent full data collection phase, 328 behaviors were coded in the three matches analyzed.

Handball matches are officiated by two referees with the same level of responsibility. In each match, the actions of the referees were coded simultaneously by three previously trained observers using the consensus agreement method. The observers were all regional-level handball referees. There are two referees in handball, a court referee and a goal-line referee, and these generally position themselves opportunely to cover critical areas of the playing court at any given time.

Polar coordinate analysis, through the calculation of Z_*sum*_ statistics derived from adjusted residuals corresponding to prospective and retrospective lags, indicates the nature of the relationship between a focal and a conditional behavior, which can be excitatory or inhibitory. The type of relationship is determined by the quadrant in which the corresponding vector is located, and the focal behavior will always be excitatory or inhibitory. The meaning of the four quadrants is shown below:

1. Quadrant I: Mutual excitation between focal and conditional behavior (i.e., prospective and retrospective activation),
2. Quadrant II: Inhibitory focal behavior and excitatory conditional behavior (i.e., prospective inhibition and retrospective activation),
3. Quadrant III: Mutual inhibition between focal and conditional behavior (i.e., prospective and retrospective inhibition),
4. Quadrant IV: Excitatory focal behavior and inhibitory conditional behavior (i.e., prospective activation and retrospective inhibition).

The following events were excluded from the analysis and were therefore not recorded as correct calls: goals, throw-offs (recorded as an error if incorrectly executed), whistle for a free throw, throw-in, or goalkeeper throw. As one of the criterion was a whistle signal by a referee, application of the advantage rule was not recorded as a correct call.

## Results

### Data quality

The correlation coefficients in Table 2 show that the *ad hoc* observation instrument allowed for the reliable and accurate recording of data.

**Table 2:** Intra and inter-observer agreement.

**Coefficient for entire session**	**Intra-observer agreement (Obs. 1 vs. Obs. 1bis)**	**Inter-observer agreement (Obs. 1 vs. Obs. 2)**
Pearson's	0.9981	0.9982
Spearman's (p)	0.9987	0.9975
Kendall's tau-b	1	1
Kappa	0.7222	0.7573
Phi	0.8345	0.8782

### Generalizability analysis

Generalizability analysis is used to estimate accuracy, validity, reliability, and sample size (Blanco-Villaseñor et al., 2014). The analysis consists of analyzing potential sources of variation that might be affecting an observational measurement or measurement design and estimating the generalizability of the design with respect to the particular conditions of a theoretical value (Blanco-Villaseñor et al., 2014).

The results for the measurement design [Criteria] [Categories]/[Observers] are shown in Tables 3, 4. The largest source of variation was associated with the interaction [Criteria] [Categories].

**Table 3:** Sources of variation, sum of squares, degrees of freedom, mean squares, %, and standard error.

**Sources of variation**	**Sum of squares**	**GC**	**Mean squares**	**%**	**Standard error**
Observers	4.675	2	2.337	0.006	0.020
Criteria	64.032	5	12.806	0.000	6.280
[Observers][Criteria]	1.516	10	0.152	0.000	0.010
Categories	2,5796.778	13	1,984.368	4.764	42.837
[Observers][Categories]	16.437	26	0.632	0.000	0.035
[Criteria][Categories]	99,194.079	65	1,526.063	95.048	87.888
[Observers][Criteria][Categories]	126.706	130	0.975	0.182	0.120

*GC, Generalizability Coefficient*.

**Table 4:** Absolute generalizability coefficient, relative generalizability coefficient, absolute SD, and relative SD in relation to measurement design.

**Measurement design**	**Absolute generalizability coefficient**	**Relative generalizability coefficient**	**Absolute SD**	**Relative SD**
[Criteria][Categories][Observers]	0.999	0.999	0.579	0.570
[Observers][Criteria][Categories]	0.001	0.001	6.181	6.032

The results of the generalizability analysis show optimal values for absolute and relative generalizability coefficient values, in addition to a linear tendency for the SDs of each design. In all cases, the relative SD was lower than the absolute SD.

### Polar coordinate analysis

**Figure 1:**
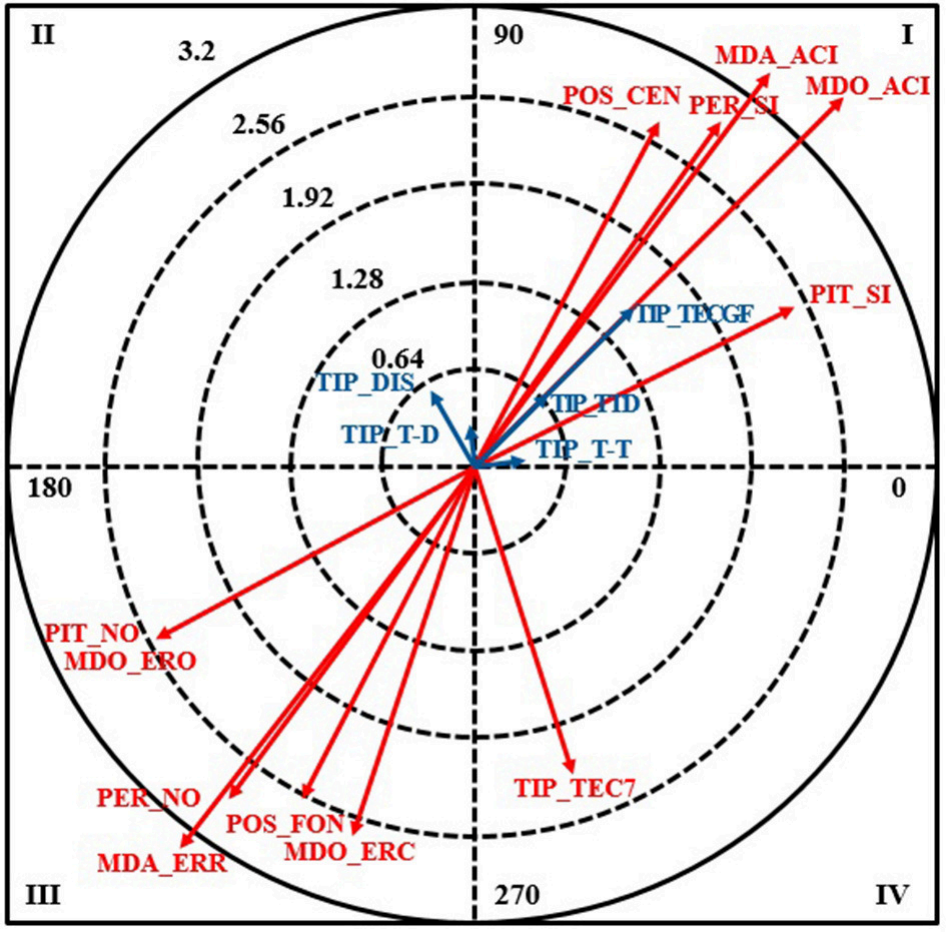
Vector map for focal behavior MDO_ACI.

The vector maps for the three focal behaviors selected for the polar coordinate analysis are shown below. The following results were obtained for MDO-ACI (correct call by right referee) (Table 5, Figure 1).

**Table 5:** Relationships between focal behavior MDO_ACI and conditional behaviors.

**Category**	**Quadrant**	**Prospective**	**Retrospective**	**Ratio**	**Radius**	**Angle**
POS_CEN	I	1.27	2.34	0.88	2.66^*^	61.42
PER_SI	I	1.81	2.38	0.8	2.99^*^	52.7
PIT_SI	I	2.21	1.27	0.5	2.55^*^	29.84
MDO_ACI	I	2.46	2.46	0.71	3.48^*^	45
TIP_TECGF	I	1.09	1.11	0.71	1.56	45.56
TIP_T-T	I	0.26	0.03	0.1	0.26	5.75
TIP_TTD	I	0.44	0.45	0.71	0.63	45.6
MDA_ACI	I	2.05	2.66	0.79	3.36^*^	52.35
TIP_DIS	II	−0.26	0.49	0.88	0.56	118.08
TIP_T-D	II	−0.06	0.42	0.99	0.42	98.19
POS_FON	III	−1.27	−2.34	−0.88	2.66^*^	241.42
PER_NO	III	−1.81	−2.38	−0.8	2.99^*^	232.7
PIT_NO	III	−2.21	−1.27	−0.5	2.55^*^	209.84
MDO_ERO	III	−2.21	−1.27	−0.5	2.55^*^	209.84
MDO_ERC	III	−0.93	−2.63	−0.94	2.79^*^	250.44
MDA_ERR	III	−2.05	−2.66	−0.79	3.36^*^	232.35
TIP_TEC7	IV	0.63	−2.2	−0.96	2.29^*^	285.94

* *p < 0.05*.

In quadrant I, the following conditional behaviors were significantly associated (>1.96) with a correct call made by the right referee (MDO_ACI): the call was made by the court referee (POS_CEN), the referee was responsible for the call (PER_SI), and the whistle was blown (PIT_SI). Relationships in quadrant I are mutually excitatory, i.e., the focal and conditional behaviors activate each other.

There were no significant relationships in quadrant II.

In quadrant III, the following behaviors were significantly associated with MDO_ACI: the call was made by the goal-line referee (POS_FON), the call was not the responsibility of the referee (PER_NO), the whistle was not blown (PIT_NO), error of omission (MDO_ERO), error of commission (MDO_ERC), and incorrect call (MDA_ERR). As expected, the focal behavior inhibited the other two categories in the same criterion, as they are mutually exclusive. It also inhibited not blowing the whistle, as for MDO-ACI to occur, the referee has to use his whistle.

In quadrant IV, just one behavior was significantly associated with MDO-ACI: TIP_TEC7 (7-meter throw foul). This shows that the likelihood of this foul being called correctly by the right referee is very high.

**Figure 2:**
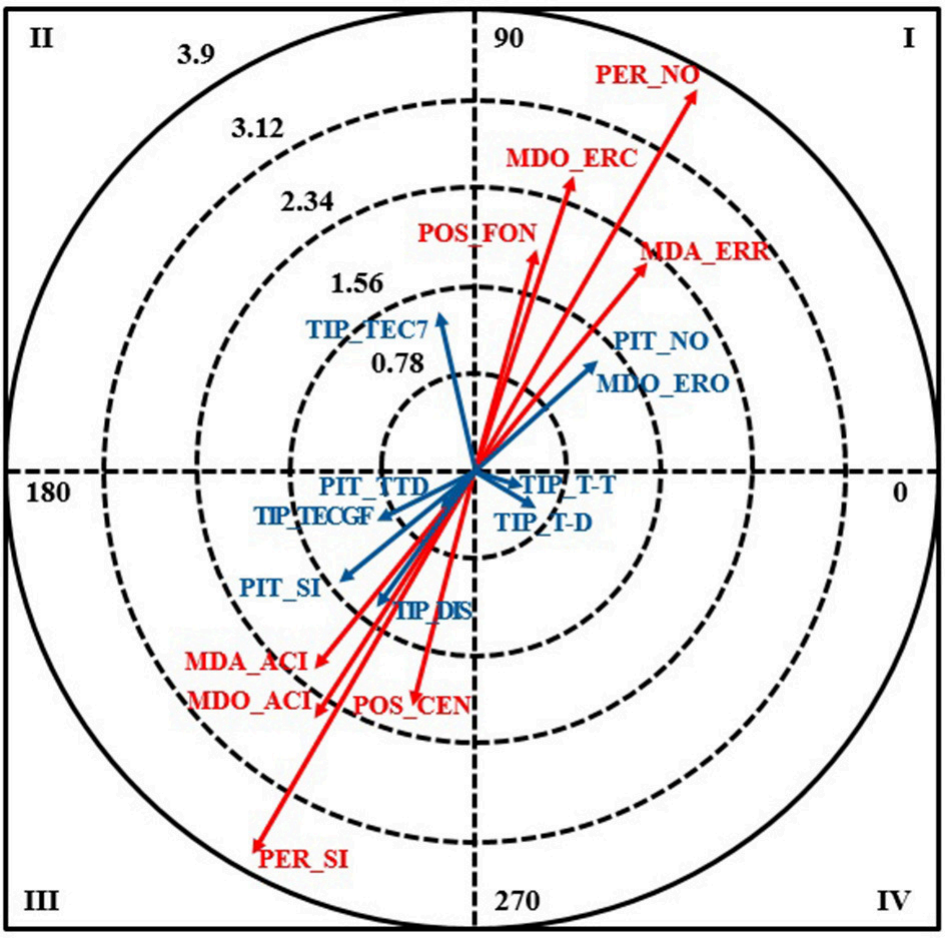
Vector map for focal behavior MDO_ERO.

The following results were obtained for the error of omission category MDO-ERO, which is when a referee should have made a call but did not (Table 6, Figure 2).

**Table 6:** Relationships between focal behavior MDO_ERO and conditional behaviors.

**Category**	**Quadrant**	**Prospective**	**Retrospective**	**Ratio**	**Radius**	**Angle**
POS_FON	I	0.47	1.92	0.97	1.97^*^	76.29
PER_NO	I	1.86	3.23	0.87	3.73^*^	60.02
PIT_NO	I	1.01	1.01	0.71	1.43	45
MDO_ERO	I	1.01	1.01	0.71	1.43	45
MDO_ERC	I	0.74	2.42	0.96	2.53^*^	72.92
MDA_ERR	I	1.38	1.78	0.79	2.26^*^	52.22
TIP_TEC7	II	−0.38	1.43	0.97	1.48	104.94
POS_CEN	III	−0.47	−1.92	−0.97	1.97^*^	256.29
PER_SI	III	−1.86	−3.23	−0.87	3.73^*^	240.02
PIT_SI	III	−1.01	−1.01	−0.71	1.43	225
MDO_ACI	III	−1.29	−2.14	−0.86	2.5^*^	238.96
TIP_TECGF	III	−0.78	−0.53	−0.56	0.94	214.35
TIP_DIS	III	−0.68	−1.03	−0.83	1.24	236.6
TIP_TTD	III	−0.19	−0.16	−0.64	0.25	219.83
MDA_ACI	III	−1.38	−1.78	−0.79	2.26^*^	232.22
TIP_T-D	IV	0.46	−0.27	−0.51	0.53	329.64
TIP_T-T	IV	0.36	−0.12	−0.33	0.38	341.03

* *p < 0.05*.

The following conditional behaviors were associated with MDO_ERO (error of omission) in quadrant I: the call was made by the goal-line referee (POS_FON), the call was not the responsibility of the referee (PER_NO), error of commission (MDO_ERC), and incorrect call (MDA_ERR). As they are located in quadrant I, the focal and conditional behaviors are mutually excitatory.

No significant relationships were detected in quadrant II or IV.

In quadrant III, the following behaviors were significantly associated with MDO_ERO: the call was made by the court referee (POS_CEN), the call was not the responsibility of the referee (PER_SI), correct call made by the right referee (MDO_ACI), and correct call (MDA_ACI). As expected for quadrant III, the focal behavior (incorrect call) inhibited correct calls.

**Figure 3:**
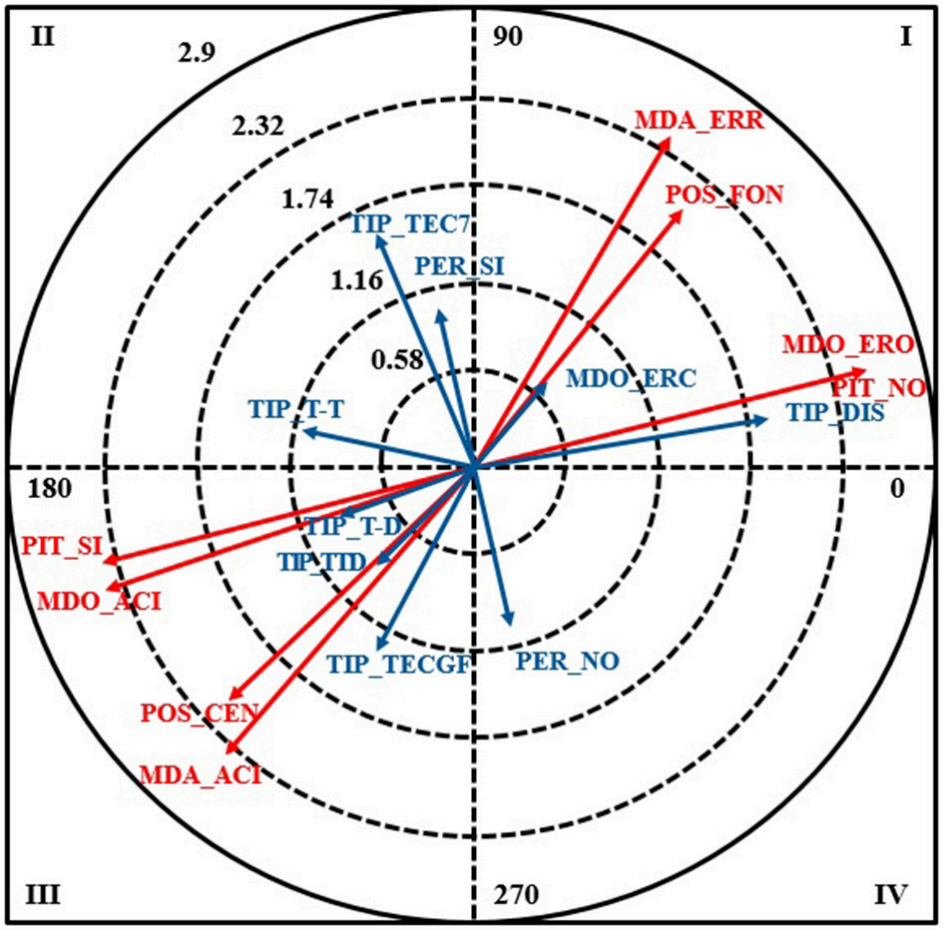
Vector map for focal behavior MDO_ERC.

The following results were obtained for the error of commission category MDO-ERC, which is when a referee made a call that was not his responsibility (Table 7, Figure 3).

**Table 7:** Relationships between focal behavior MDO_ERO and conditional behaviors.

**Category**	**Quadrant**	**Prospective**	**Retrospective**	**Ratio**	**Radius**	**Angle**
POS_FON	I	1.5	1.51	0.71	2.13^*^	45.3
PIT_NO	I	2.42	0.74	0.29	2.53^*^	17.08
MDO_ERO	I	2.42	0.74	0.29	2.53^*^	17.08
MDO_ERC	I	0.47	0.47	0.71	0.66	45
TIP_DIS	I	1.74	0.4	0.22	1.78	12.99
MDA_ERR	I	1.39	1.95	0.81	2.4^*^	54.49
PER_SI	II	−0.2	0.95	0.98	0.97	101.78
TIP_T-T	II	−1.1	0.28	0.25	1.13	165.73
TIP_TEC7	II	−0.62	1.49	0.92	1.61	112.45
POS_CEN	III	−1.5	−1.51	−0.71	2.13^*^	225.3
PIT_SI	III	−2.42	−0.74	−0.29	2.53^*^	197.08
MDO_ACI	III	−2.41	−0.91	−0.35	2.57^*^	200.66
TIP_TECGF	III	−0.61	−1.14	−0.88	1.3	241.88
TIP_T-D	III	−0.79	−0.3	−0.36	0.84	200.91
TIP_TTD	III	−0.58	−0.6	−0.72	0.83	226.04
MDA_ACI	III	−1.39	−1.95	−0.81	2.4^*^	234.49
PER_NO	IV	0.2	−0.95	−0.98	0.97	281.78

* *p < 0.05*.

The following conditional behaviors were all significantly associated with the focal behavior in quadrant I: goal-line position (POS_FON), whistle not blown (PIT_NO), and error of omission (MDO_ERO).

No significant relationships were detected in quadrant II or IV.

MDO_ERO was significantly associated with several behaviors in quadrant III: central-court position (POS_CEN), whistle blown (PIT_SI), correct call made by right referee (MDO_ACI), and correct call (MDA_ACI). Again, the focal behavior inhibited behaviors related to correct calls.

## Discussion

We have presented a new tool for observing, coding, and analyzing the actions of referees in handball competitions. Although decisions made by referees are influenced by contextual factors (Debanne, 2014), the *ad hoc* observation instrument described in this study was designed to provide an objective means of recording, describing, and analyzing actions taken by handball referees according to their role and position on the court. While observational methodology has been used to analyze handball, studies to date have focused on game situations from the players' perspective (González et al., 2013; Sousa et al., 2014).

The reliability, generalizability, and correlation results in the data quality control analysis attest to the suitability of the data obtained. The observation instrument thus would appear to be an adequate tool for obtaining reliable datasets for performing sequential and other analyses of the performance of court and goal-line referees during handball competitions. In this respect, it is similar to observation instruments designed for other sports, such as soccer (Sarmento et al., 2010), basketball (Garzón et al., 2011), waterpolo (Santos et al., 2014), and beach handball (Morillo and Hernández-Mendo, 2015). A recent study by Araujo et al. (2016) addressed the issue of decision-making in sport and argued that the use of observation to analyze specific actions and behaviors could provide complementary insights into this complex process. The conceptual vector maps presented in this study show how the referees responded to events based on their use of the whistle. The instrument presented has numerous applications. It could be used, for example, to identify streams of behavior or specific actions that cause greater difficulties for referees or situations that are prone to more error, regardless of level of physical fitness. Handball refereeing has been reported to require moderate levels of fitness and does not appear to be limited by aerobic capacity (Fernandes da Silva et al., 2010).

Most of the correct calls were made from the central court position. This is logical, as court referees are generally responsible for making more calls than goal-line referees and have to deal with less conflictive situations. Goal-line referees, by contrast, have to deal with multiple interactions in short spaces of time and are therefore more likely to make incorrect calls, even though they use their whistle less. We also found that the referees observed made a high percentage of correct calls. Seven-meter throw fouls, for example, were correctly called by the right referee (the goal-line referee) in all cases. This again is logical, as fouls of this type are generally the responsibility of the referee at the end of the court and are rarely called by the court referee.

Handball, unlike other sports such as basketball, does not use instant-replay or similar technology to facilitate the work of referees. The installation of court-side cameras to watch instant or near-instant replays of dubious play or the use of goal-line sensors to check whether or not the ball completely crossed the goal-line could lead to interesting improvements in the game. Such measures, however, also have drawbacks. The technology is costly and perhaps should only be considered for elite competitions. In addition, the use of these systems could hurt the credibility of referees and cause them to lose confidence in their calls, particularly in the case of less experienced referees. Novice referees have been found to perform less well than “expert referees” with greater knowledge, experience, and expert memory (Abdeddaim et al., 2016).

More studies of decision-making by handball referees are needed to assess the possible advantages of redistributing responsibilities and zones between both referees and even perhaps of using a third referee in areas with high error rates. Our study highlights some limitations that could be overcome in future studies. It would be interesting, for example, to analyze more areas of the court and to divide the court into specific zones to analyze the actions of referees according to the number of players on the court at a given time and the position of the defense. The distribution of responsibilities is more complicated in open and man-to-man defences, as it is less clear in such cases who is responsible for calling what. It is also complicated to determine whether a referee chose not to make a call or decided to apply the advantage rule, as there are no official hand signals for this decision. The rules do, however, specify that referees should refrain from interrupting the game prematurely to allow continuity of play where possible. Accordingly, there may be some overlap between application of the advantage rule and errors of omission.

Although some research has already been done on how player gender can influence decision-making by referees in handball (Souchon et al., 2010), more work in this area is necessary. Finally, it would be interesting to analyze different championships over time to monitor the influence of new rules and regulations and changes in refereeing practice and performance.

## Author contributions

AH, VM and RR: design of the work; acquisition, analysis, and interpretation of data for the work. JM: acquisition, analysis, and interpretation of data for the work; AM: acquisition and analysis of data for the work. All authors: Drafting the work or revising, final approval of the version and agreement to be accountable for all aspects of the work.

### Conflict of interest statement

The authors declare that the research was conducted in the absence of any commercial or financial relationships that could be construed as a potential conflict of interest. The reviewer, JT, declared a shared affiliation, though no other collaboration, with the authors to the handling Editor.
